# Genomic profiling of AKT1 and NRAS mutations using NGS in Egyptian hepatocellular carcinoma

**DOI:** 10.1007/s12672-025-04015-8

**Published:** 2025-11-26

**Authors:** Magdy M. Mohmed, Randa M. Talaat, Moustafa A. Sakr, Mohamed K. Khalifa, Mofida A. Keshk, Ehab A. Ahmed, Mohmed A Selim, Sawsan M. Abdelmonam, Ghada M. Nasr, Manal O. El Hamshary

**Affiliations:** 1 Department of Molecular Diagnostics and Therapeutics, Genetic Engineering and Biotechnology Research Institute (GEBRI), University of Sadat City, Sadat City, 32897 Egypt; 2 Department of Molecular Pathology, Children Cancer Hospital 57357, Cairo, Egypt; 3 Omicsense Company, Cairo, 12311 Egypt; 4 Chemistry Department, Faculty of Science, Medical Genome Center Faculty of Medicine, Cairo University, Cairo, Egypt; 5 Botany and Microbiology Department, Faculty of Science (boys), AL-Azhar University, Cairo, Egypt; 6 Professor and Ex-Head of Hepato-Gastroenterology, Mansoura Specialized Hospital, Mansoura University, Mansoura, Egypt

**Keywords:** AKT1, NRAS, PI3K/AKT signaling pathway, HCC, NGS, Somatic mutations

## Abstract

**Background and objectives:**

Hepatocellular carcinoma (HCC) remains a major health burden in Egypt, with underlying molecular mechanisms still poorly defined. This study aimed to characterize somatic mutations in AKT1 and NRAS genes using targeted next-generation sequencing in Egyptian HCC patients.

**Methods:**

Cell Tumor DNA (ct DNA) from 50 patients, divided into 25 HCC patients, were classified according to Barcelona clinic Liver Cancer (BCLC) staging alongside HCV-infected and normal controls, was analyzed using the Ion AmpliSeq Cancer Hotspot Panel. Mutational profiles were verified using the Integrative Genomics Viewer and functionally annotated via different cancer databases.

**Results:**

A total of 16 somatic mutations were identified (12 in AKT1, 4 in NRAS). The most frequent AKT1 mutation was p.Asp32Glu, observed in multiple clinical groups. Other variants, including p.Glu40Gly and p.Asn31Ile, affected conserved regions of the kinase domain. AKT1 mutations were more prevalent in intermediate and advanced HCC stages, suggesting a role in tumor progression. In contrast, NRAS mutations were rare and evenly distributed across stages, with limited clinical correlation. Functional predictions supported the potential pathogenicity of several AKT1 and NRAS variants.

**Conclusion:**

This study highlights a higher frequency and diversity of AKT1 mutations in Egyptian HCC patients, particularly in progressive disease stages, supporting their potential role in HCC development and progression. These findings advocate for expanded molecular profiling in HCC and suggest AKT1 as a promising target for personalized therapy.

## Introduction

Hepatocellular carcinoma (HCC) is the most common primary malignancy of the liver, accounting for nearly 90% of all primary liver malignancies and occurring in many of the world’s most populous regions. In 2018, Egypt had the second highest prevalence of liver cancer, with chronic liver disease accounting for more than 90% of all HCC cases. Cirrhosis, regardless of origin, is the leading risk factor for HCC [1].

Viral infections, hereditary susceptibility, hepatotoxic drug exposure, cigarette use, alcoholism, metabolic syndrome, and liver cancer are all risk factors for HCC [2]. Cirrhosis develops in nearly all cases of hepatitis C virus (HCV) infection (93%) [3]. In Egypt, 20% of HCV patients developed cirrhosis, becoming the nation the most HCV-endemic globally [4].

Individuals identified as being at elevated risk for HCC development, including those with cirrhosis or infections with hepatitis B or C, necessitate prompt detection of HCC, as indicated by research findings. Alpha-fetoprotein (AFP) levels were incorporated into the standard biochemical assessments. In recent years, cell-free DNA (cfDNA) has emerged as a potential biomarker of interest. Biomarkers for cancer diagnosis, prognosis, and therapy can be identified in peripheral blood, which serves as a convenient source of cell-free DNA (cfDNA). The potential applications of ctDNA in cancer therapy are garnering significant attention. We explore the potential application of ctDNA analysis in clinical oncology with a focus on Egyptian patients for purposes such as cancer screening, early diagnosis, therapy evaluation, disease progression monitoring, and prognosis assessment. Similar studies were performed by [5, 6] on members of the Chinese population.

RAS is a proto-oncogene belonging to the Ras family located on chromosome 1. It produces a tiny GTPase that facilitates intercellular signaling. Its mutations are associated with several cancers, including hepatocellular carcinoma (HCC), wherein the RAS/MEK/ERK pathway facilitates tumor proliferation by activating NRAS [7]. The human AKT1 gene, a proto-oncogene variant of the viral v-akt, is located on chromosome 14q32, in proximity to the IGHM locus. This gene encodes a significant kinase within the PI3K/Akt signaling pathway, which regulates essential cellular functions such as growth, survival, and metabolism. If this route is disrupted, it can disturb the equilibrium of cells and has been associated with liver inflammation, fibrosis, and a poor prognosis for individuals with HCC [8, 9]. Prior investigations into the roles of AKT and RAS in liver cancer progression have demonstrated that just activation of AKT is insufficient for rapid tumor proliferation; hepatocellular carcinoma (HCC) requires over 30 weeks to develop [10].

This research aimed to identify the mutational spectrum of the NRAS and AKT1 genes in Egyptian patients with HCC and to assess the impact of these mutations on prognosis and responsiveness to targeted treatment.

## Materials and methods

### Study design and participants

A total of 50 participants were enrolled in this study, including 25 healthy controls, 21 patients diagnosed with hepatocellular carcinoma (HCC), and 4 patients with chronic HCV infection without malignancy. Two peripheral blood samples were collected from each participant on the same day to obtain an adequate quantity of cfDNA for reliable molecular analysis. The average age of the patients was 62 years; 21 individuals were diagnosed with HCC, whereas 4 individuals had HCV without development to malignancy. Informed consent was obtained from all individual participants included in this study. At the National Liver Institute in Menoufia, Egypt, we solely enrolled patients with comprehensive medical records, confirmed diagnoses, and completed treatment protocols. Patients were eligible for inclusion if they had a histologically or radiologically confirmed diagnosis of primary hepatocellular carcinoma (HCC) at any clinical stage, according to established diagnostic guidelines. Both viral-related HCC (associated with hepatitis B or C infection) and non-viral HCC cases were included to ensure comprehensive representation of the major etiological subtypes of the disease. Other cancer patients were excluded from this investigation.

All patients with family history underwent a wide range of diagnostic tests, including a chest X-ray, a thorough laboratory panel (including liver enzymes, coagulation profile, renal function profile, and complete blood count), tumor staging, and a comprehensive clinical examination. During the course of the experiment, no further cancer patients were added. The Egyptian Liver Institute’s oncologists and radiologists classified the included patients according on their diagnostic and staging status.

### Sample collection and cell-free DNA extraction

Whole blood samples (1–3 mL) were collected in EDTA tubes and genomic DNA extracted from them. To retain the nucleic acids in circulation, plasma was separated after collection and stored at -80 °C. We next used the QIAamp^®^ DSP Virus Spin Kit, Version 1 (QIAGEN, Hilden, Germany) to extract cell-free DNA (cfDNA) from plasma according to the manufacturer’s instructions.

### Primer design

This investigation was facilitated by the use of primers from the Ion AmpliSeq Cancer Hotspot Panel v2 (primer pool) (catalog number 4475346, Thermo Scientific, USA). The NGS technique was performed utilizing Ion Torrent Next Generation Sequencing.

### Library Preparation

The library was developed using the Ion AmpliSeq Library kit 2.0 (catalogue number 4475345, Thermo Scientific, USA). The kit enabled the amplification, partial digestion, ligation of the adaptor and barcodes, as well as the purification and quantification processes.

### Template Preparation

The template was prepared using the Thermo Scientific, USA-based Ion PGMTM Hi-QTM View OT2 kit (catalog number A29900). We utilized both Ion OneTouchTM 2 and Ion OneTouchTM ES procedures to prepare the template.

### Sequencing

The sequencing was conducted using the Ion PGMTM Hi-QTM View Sequencing kit (catalogue number A30044, Thermo Scientific, USA).

### Data analysis

**Fig. 1:**
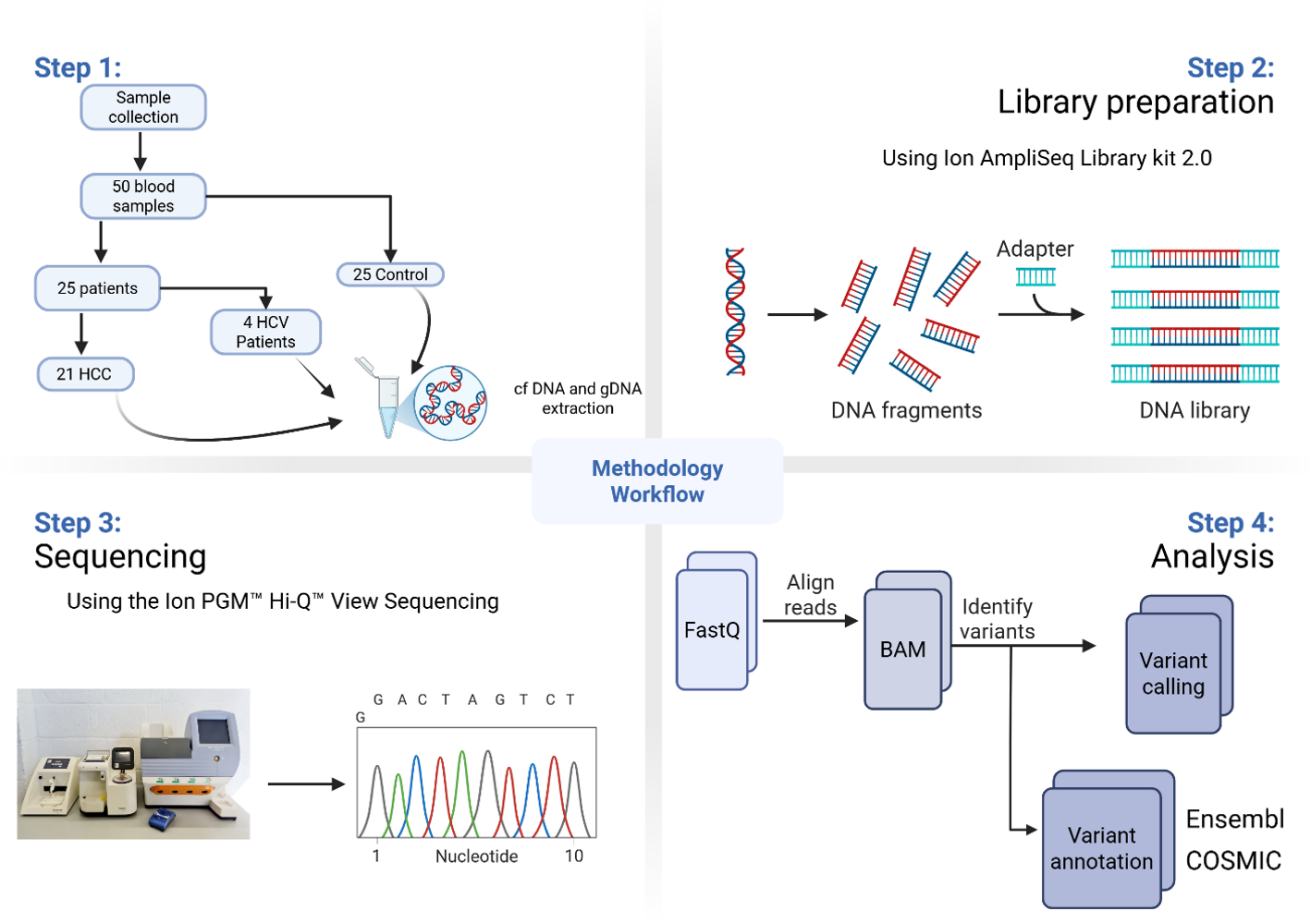
workflow of study methodology

For analysis, the BAM files of matched normal and tumor samples were uploaded to the cloud-based Ion Reporter server (version 5.10) on the Thermo Fisher website. The standard plugin parameters and the ion AmpliSeq hotspot panel protocol were implemented. The data was aligned to Human Genome version 19 (hg19) using Torrent Suite (version 3.6.2; Thermo Fisher Scientific, Inc.). The coverage analysis was conducted using the Coverage Analysis plug-in (version 3.6; Thermo Fisher Scientific, Inc.). It was determined that cases with a frequency of 10%, an average base coverage of 500X readings, and a quality of 20% did not provide any valuable information. The Variant Caller plug-in (version 3.6; Thermo Fisher Scientific, Inc.) was used to corroborate each mutation, and the Integrative Genome Viewer (IGV) from the Broad Institute (www.broadinstitute.org) was employed to identify each mutation. The generated variants were analyzed using a variety of databases. Ensembl (https://asia.ensembl.org/index.html) and the COSMIC database, a “Catalogue of Somatic Mutations in Cancer” (http://cancer.sanger.ac.uk/cosmic), were the databases. An integrated overview of the methodology and analysis pipeline is provided in Fig. 1.

## Results

Fifty Egyptians participated in the study; twenty-one had HCC, four had HCV but never had cancer, and twenty-five were deemed normal. Patients with complete medical histories who underwent treatment at the National Liver Institute’s Menoufia facility were eligible for inclusion in the research. Clinicopathological variables, patient characteristics, and outcomes were all evaluated, and BCLC staging were summarized as shown in Tables 1 and 2.

**Table 1:** Demographic and clinical characteristics of HCC patients (*n* = 21)

Variable	Category	No.	%
Age (years)	< 60	8	38.1
	≥ 60	13	61.9
Sex	Female	3	14.3
	Male	18	85.7
Smoking status	Current smoker	3	14.3
	Non-smoker	14	66.7
	Ex-smoker	4	19.0
History of Bilharzia	Yes	13	61.9
Family history of HCC	Yes	4	19.0
Comorbidities	DM	7	33.3
	HTN	3	14.3
Viral infection	HCV	18	85.7
	HBV + HCV	1	4.8
	NBNC	2	9.5

NBNC = no B no C, DM: Diabetes Mellitus, HTN: Hypertension, HCV: hepatitis C virus, HBV: hepatitis B virus

**Table 2:** Tumor and laboratory characteristics of HCC patients (*n* = 21)

Variable	Category	No.	%
AFP	≤ 20 ng/mL	6	28.6
	> 20 ng/mL	15	71.4
Ascites	None	17	81.0
	Minimal	3	14.3
	Moderate	1	4.7
Portal vein invasion	Present	3	14.3
Lymph node metastasis	Present	3	14.3
Lung metastasis	Present	3	14.3
Child-Pugh classification	A	16	76.2
	B	3	14.3
	C	2	9.5
Tumor characteristics	Single lesion	10	47.6
	Multiple lesions	11	52.4
	Tumor size < 3 cm	5	23.8
	Tumor size > 3 cm	16	76.2
BCLC stage	A	7	33.3
	B	5	23.8
	C	7	33.3
	D	2	9.5

AFP: Alpha fetoprotein, BCLC: Barcelona clinic liver cancer

Variant Caller plug-in (version 3.6; Thermo Fisher Scientific, Inc.) was used and mutations were detected. Using the Integrative Genome Viewer (IGV) from the Broad Institute (www.broadinstitute.org) each mutation was verified.

**Fig. 2:**
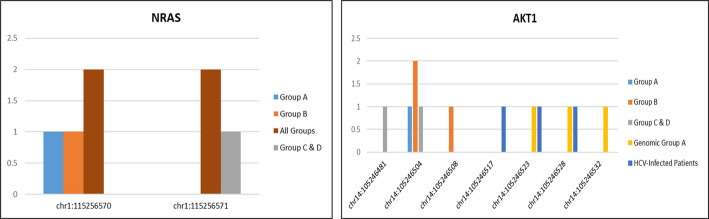
The frequency of each mutation in different groups. each mutation was plotted according to the number of candidates that have this mutation and in which group

**Fig. 3:**
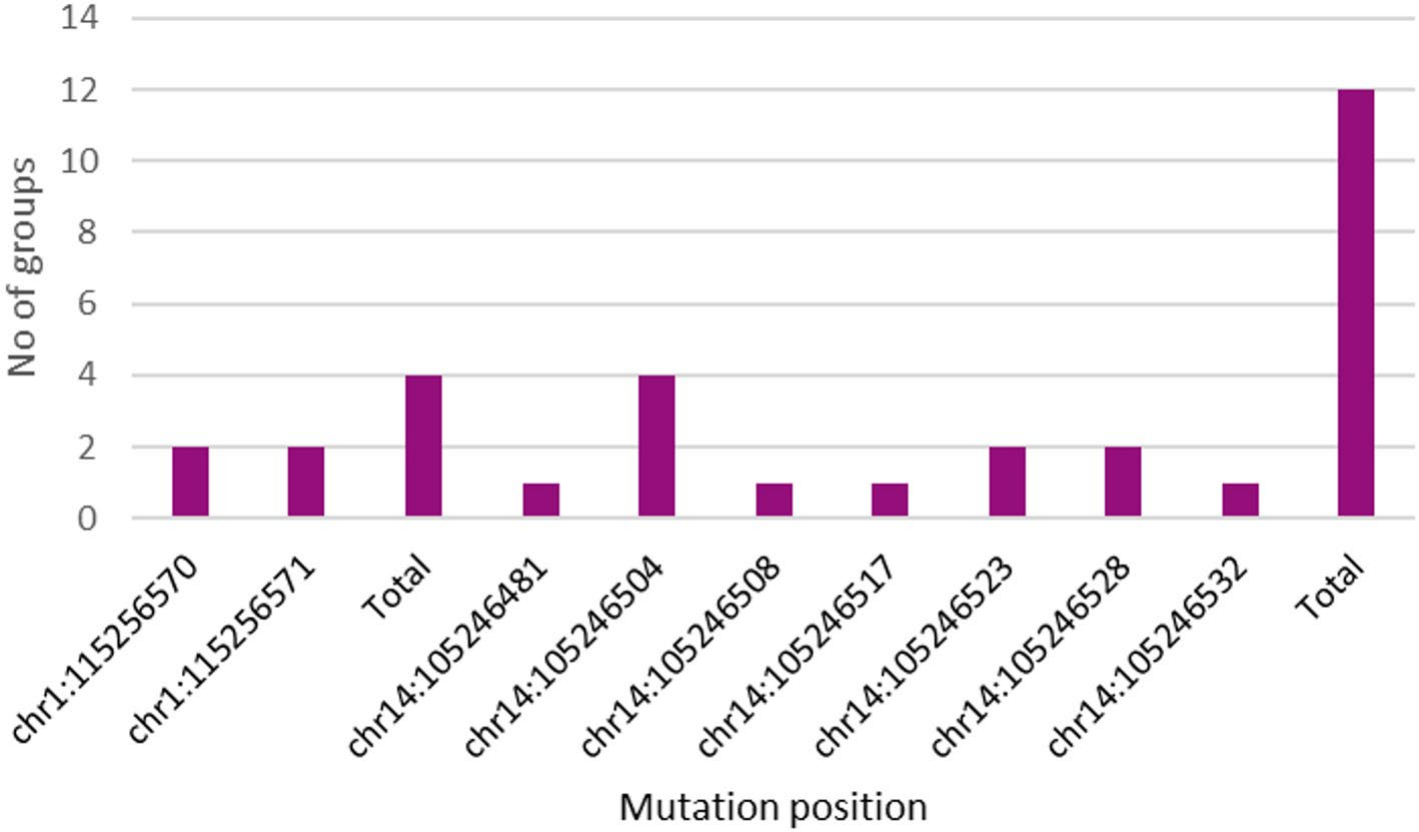
The frequency of each mutation in respect to the total number of samples having this mutation in all groups

In a study of HCC patients, two unique NRAS mutations were identified at the chr1:115256570 locus, leading to an amino acid alteration. One member of Group A and one member of Group B possessed the p.Asp32Glu (c.141T > A) variation. The p.Asp32Ala (c.140 A > C) mutation was identified at chr1:115256571 in a patient from Groups C and D, as well as in an individual with HCV infection. It is important to emphasize that none of these NRAS mutations were identified in the specified chromosomal groupings. Four distinct mutation events impacting the NRAS gene were identified across different clinical cohorts, albeit none were situated within the same genomic cluster. Eight unique variations of AKT1 were identified over 12 occurrences. The most common variant was p.Asp32Glu (c.96T > A), identified in four individuals from Groups A, B, C, and D. Additional non-synonymous alterations included p.Glu40Gly (c.119 A > G), p.Asn31Ile (c.92 A > T), p.Leu28Pro (c.83T > C), p.Tyr26Cys (c.77 A > G), and p.Arg23Leu (c.68G > T). It is essential to emphasize that both HCV-infected individuals and those belonging to genomic Group A possess the synonymous mutations p.Pro24= (c.72 A > T) and p.Tyr26Cys. AKT1 mutations were more common than NRAS mutations overall and were identified in all clinical groups, including three instances in genomic Group A. Details of mutations in patients of stage A, B, and late group are illustrated in Table 3 as well as Figs. 2 and 3.

**Table 3:** Distribution of NRAS and AKT1 gene mutations across clinical and genomic subgroups in HCC patients

Gene	Mutation	Amino acid change	cDNA change	Group A	Group B	Group C & D	Genomic group A	Genomic group B	Genomic group C & D	HCV-infected patients	All groups
NRAS	chr1:115256570	p. Asp47Glu	c.141T > A	1	1	-	-	-	-	-	2
	chr1:115256571	p. Asp47Ala	c.140 A > C	-	-	1	-	-	-	1	2
	**Total**			1	1	1	0	0	0	1	4
AKT1	chr14:105246481	p.Glu40Gly	c.119 A > G	-	-	1	-	-	-	-	1
	chr14:105246504	p.Asp32Glu	c.96T > A	1	2	1	-	-	-	-	4
	chr14:105246508	p.Asn31Ile	c.92 A > T	-	1	-	-	-	-	-	1
	chr14:105246517	p.Leu28Pro	c.83T > C	-	-	-	-	-	-	1	1
	chr14:105246523	p.Tyr26Cys	c.77 A > G	-	-	-	1	-	-	1	2
	chr14:105246528	p.Pro24=	c.72 A > T	-	-	-	1	-	-	1	2
	chr14:105246532	p.Arg23Leu	c.68G > T	-	-	-	1	-	-	-	1
	**Total**			**1**	**3**	**2**	**3**	**0**	**0**	**3**	**12**

**Fig. 4:**
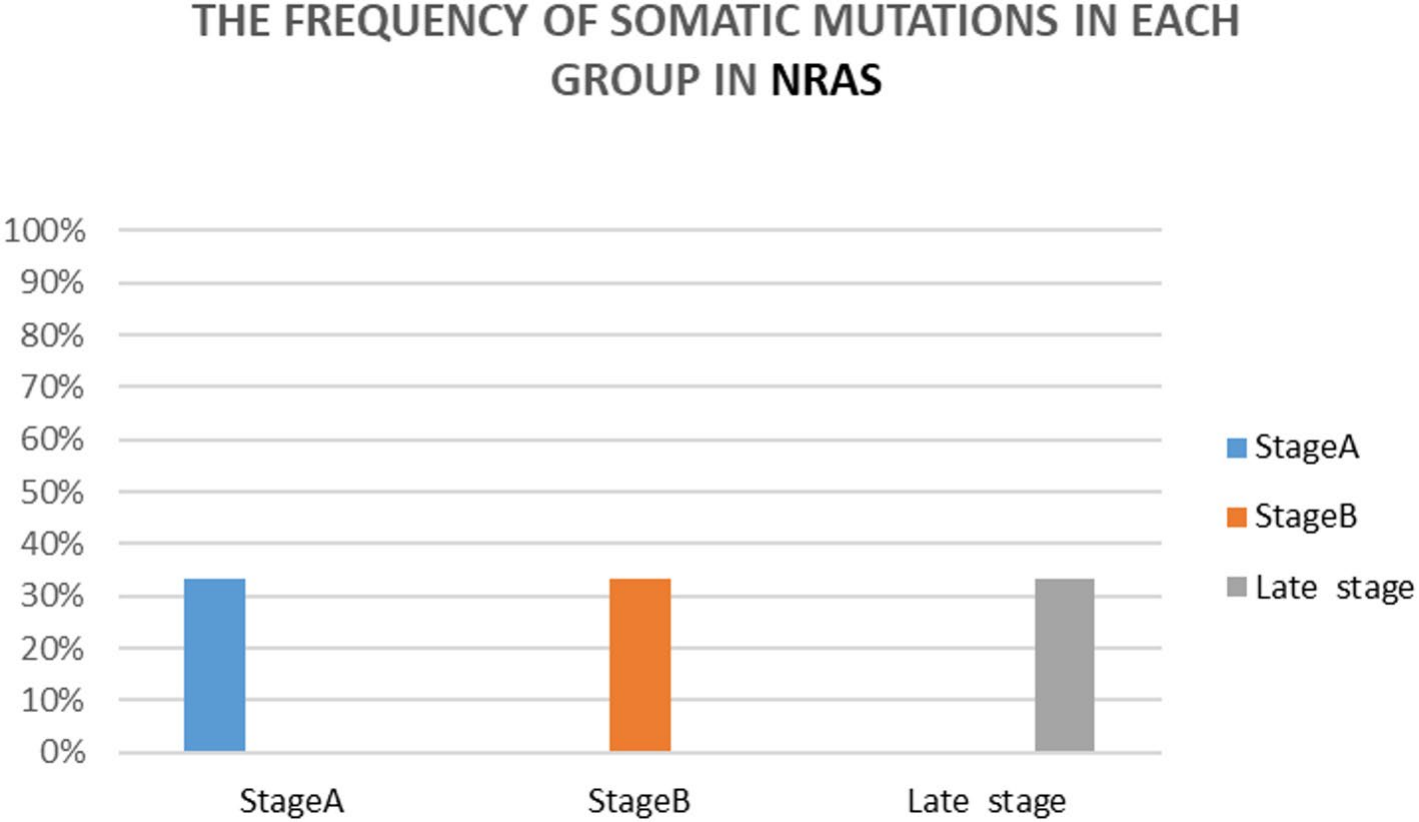
The frequency of somatic mutations in each group with respect to the total somatic mutations in NRAS

NRAS somatic mutations were identified in all clinical stages, with 33.3% detected in each of Groups A, B, and C & D (Fig. 4). The uniform distribution indicates an absence of a clear correlation between the occurrence of NRAS mutations and the progression stage of HCC in this cohort.

**Fig. 5:**
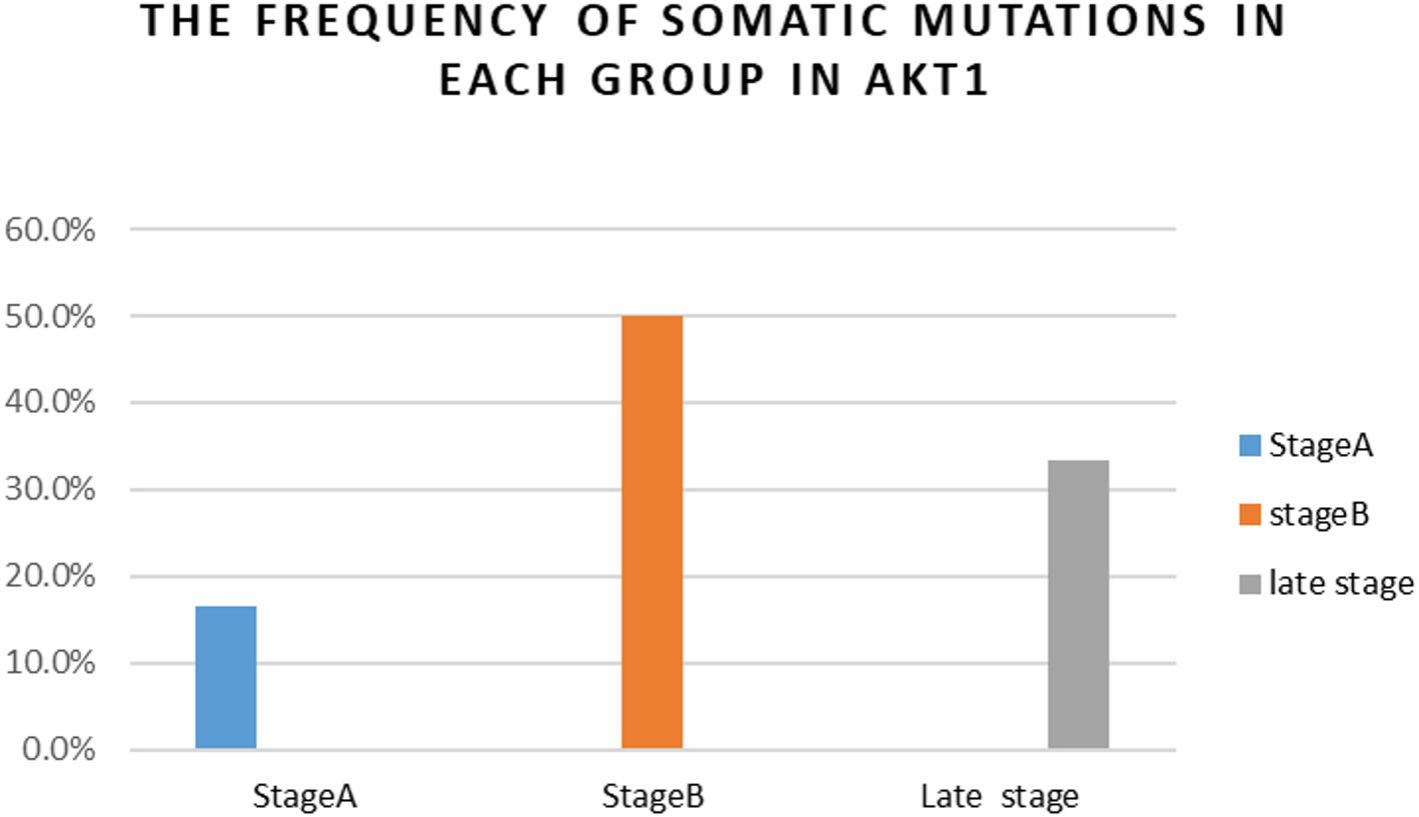
The frequency of somatic mutations in each group with respect to the total somatic mutations in AKT1

Figure 5 indicates that the prevalence of AKT1 somatic mutations was 16.7% in Group I (stage A), 50% in Group II (stage B), and 33.3% in Group III (late stage). This distribution suggests that AKT1 mutations are more prevalent in the intermediate and advanced phases of HCC, indicating a potential role of AKT1 in tumor proliferation.

Table 4 reveals that two variants in the NRAS and AKT1 genes were discovered that are unlikely to have a significant impact on how the genes function. One patient’s (HCC-21) NRAS gene contained a synonymous single nucleotide variant (p.Asp47=, chr1:115256570, GAT > GAC). This mutation does not affect the amino acid sequence, however it may have an effect on splicing or regulation. It was discovered in exon 3 (codon 141), was expected to have a little influence, and is included in the dbSNP database as rs901561299.

A non-synonymous SNV (p.Asp32Gly, chr14:105246504, GAT > GAC) was discovered in the AKT1 gene of patient HCC-9. This variant occurs in exon 4 (codon 395) and alters one amino acid at a conserved site. Based on computational scores (such as SIFT and CatCod), it is expected to have a minor impact on function. In dbSNP, it is identified as rs201636005.

**Table 4:** Synonymous and low-impact missense variants in NRAS and AKT1 identified in HCC patients

Gene	No	Chromosome	Type	Allele	AA change	Exon	CDs	Codons	Impact	SIFT CAT	Cat Cod	Link
NRAS	HCC-21	Chr1: 115,256,570	SNV	A/G	Asp47=	3/7	141	GAT/GAC	low	NA	11.99	*rs901561299*
AKT1	HCC-9	Chr14: 105,246,504	SNV	A/G	Asp32	4/15	395	GAT/GAC	Low	NA	0.902	rs201636005

### Mutational spectrum and in Silico prediction of AKT1 and NRAS variants

There were seven somatic mutations in the AKT1 and NRAS genes in the HCC patients (Table 5).

Six distinct variants of the AKT1 gene were discovered. Three patients (HCC-2, HCC-3, and HCC-5) carried the most prevalent variation, p.Asp32Glu (chr14:105246504). SIFT has always said that this repeating mutation is deleterious, and PolyPhen-2 believes it is probably hazardous. Overall, it was classed as a modifier with a moderate effect (rs201636005). There were also additional variants, such as p.Asn3Ile (HCC-4), which had contradictory predictions—SIFT indicated it was detrimental, but PolyPhen-2 stated it was harmless—and was expected to have a little impact on function. SIFT felt the p.Glu40Gly variant (HCC-15) would be OK, whereas PolyPhen-2 thought it would be detrimental. It also came into the moderate impact category.

Two alterations were identified at Codon 47 in the NRAS gene. The p.Asp47Glu mutation (chr1:115256570), perhaps associated with patient HCC-21, is considered deleterious, certainly damaging, and pathogenic (rs901561299). SIFT indicated that the second variant, p.Asp47Ala (chr1:115256571, HCC-20), was deleterious, whereas PolyPhen-2 classified it as benign. FATHMM-MKL (rs1383166562) classified it as pathogenic.

The collective data indicate a significant presence of missense mutations in conserved regions of both genes. In this HCC cohort, AKT1 p.Asp32Glu appears to be a potential hotspot.

**Table 5:** Functional annotation of NRAS and AKT1 variants identified in HCC patients

Gene	Patient ID	Chromosome	Variant type	Ref	Alt	Amino acid change	AA name	SIFT	PolyPhen-2	Impact	Cat Cod	MutationTaster	FATHMM-MKL	Codon Change	dbSNP ID
AKT1	HCC-2	chr14:105246504	SNV	A	C	p.Asp32Glu	Glu	Deleterious (0.05)	Probably damaging (0.96)	Modifier	14.35	Uncertain	Benign	GAT → GAG	rs201636005
AKT1	HCC-3	chr14:105246504	SNV	A	T	p.Asp32Glu	Glu	Deleterious (0.05)	Probably damaging (0.96)	Modifier	14.35	Uncertain	Benign	GAT → GAG	rs201636005
AKT1	HCC-4	chr14:105246508	SNV	T	A	p.Asn3Ile	Ile	Deleterious (0.02)	Benign (0.94)	Moderate	13.42	Benign Moderate	Benign	AAT → ATT	rs780173607
NRAS		chr1:115256570	SNV	A	T	p.Asp47Glu	Glu	Deleterious (0.05)	Possibly damaging (0.538)	Modifier	23.5	Disease-causing	Uncertain	GAT → GAA	rs901561299
AKT1	HCC-5	chr14:105246504	SNV	A	T	p.Asp32Glu	Glu	Deleterious (0.05)	Probably damaging (0.96)	Low	13.42	Uncertain	Benign	GAT → GAA	rs201636005
AKT1	HCC-15	chr14:105246481	SNV	T	C	p.Glu40Gly	Gly	Tolerated (0.06)	Possibly damaging (0.597)	Moderate	25.2	Uncertain	Uncertain	GAG → GGG	rs2140949199
NRAS	HCC-20	chr1:115256571	SNV	T	G	p.Asp47Ala	Ala	Deleterious	Benign (0.239)	Modifier	24.4	Disease-causing	Pathogenic Supporting	GAT → GCT	rs1383166562

**Fig. 6:**
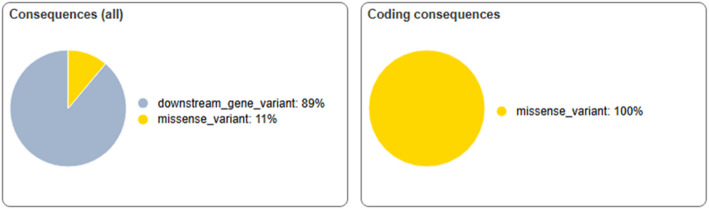
Percentage distribution of NRAS variants by all consequences and coding consequences as predicted by VEP

Using the Variant Effect Predictor (VEP) on the Ensembl platform, all of the NRAS variations were found to be missense mutations, which means they impacted the gene’s function. 89% of the identified variants were situated downstream in genes, with 11% being missense variations (Fig. 6).

**Fig. 7:**
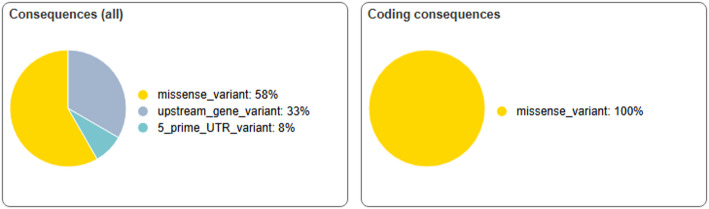
Percentage distribution of AKT1 variants by all consequences and coding consequences as predicted by VEP

Similarly, all coding area alterations in the AKT1 gene were identified as missense mutations. Missense variations accounted for 58% of the identified results, followed by upstream gene variants (33%), and 5′ UTR variants (8%). Figure 7 depicts the distribution of cumulative variation effects across both genes.

## Discussion

The purpose of this study was to gain a better knowledge of hepatocellular carcinoma (HCC) biology and the genetic complexities of HCC patients by studying the genomes of Egyptian persons with HCC. The Ion AmpliSeq Cancer Hotspot Panel NGS for tumour profiling focuses on a predetermined gene panel. These panels include genes known to be involved in HCC, allowing for the simultaneous detection of all genetic variations that might be related with HCC [11, 12].

In the present study, 85.7% of HCC patients were male, with a mean age of 62.19 ± 9.08 years. Individuals aged 60 years and older were the majority age group, accounting for 61.9% of the cohort, consistent with other global research. The findings may be ascribed to a confluence of behavioral risk factors, including elevated alcohol intake and smoking rates, alongside biological differences, such as the impact of sex hormones and disparities in immunological response [13].

Approximately 61.9% of the individuals in this study had a history of Bilharziasis (95% confidence interval: 0.384–0.819), whereas 90.5% of the HCC patients tested positive for HCV infection (95% confidence interval: 0.696–0.988). These data support prior comments that chronic hepatitis C is a substantial risk factor for hepatocellular carcinoma in Egypt [14, 15].

In this research, 33.3% of patients with hepatocellular carcinoma had diabetes, and 14.3% had hypertension. These findings support previous research demonstrating that those with type 2 diabetes and chronic liver disease are more likely to develop hepatocellular carcinoma than those with liver disease alone. People with diabetes were nearly twice as likely to acquire HCC as those without diabetes [16]. Furthermore, essential hypertension has been associated with increased mortality in patients with hepatocellular carcinoma [17].

Elevated tumor-associated lymphatic vessel density has been identified as a strong prognostic indicator of poor outcomes in hepatobiliary cancers, including HCC, emphasizing the pivotal role of lymph angiogenesis in tumor progression. This observation, as reported by [18] complements our findings by suggesting that both genetic polymorphisms and microenvironmental remodeling contribute to HCC aggressiveness and clinical prognosis.

The AKT and NRAS genes are associated with somatic mutations in individuals with and without diabetes. These mutations can modify cellular activity and may be linked to several disorders [19]. Studies demonstrate that diabetes mellitus elevates the risk of hepatocellular carcinoma, particularly in conjunction with hepatitis B and C infections. Diabetes mellitus serves as a specific risk factor for the development of hepatocellular carcinoma, independent of other liver diseases [20].

### NRAS and AKT1 mutational profiles in HCC

In this HCC cohort, 16 somatic mutations were found in NRAS (*n* = 4) and AKT1 (*n* = 12), with different distribution patterns across clinical and genomic categories.

Two NRAS variations, p.Asp47Glu and p.Asp47Ala, were identified across clinical groups but not in genomic subgroups. One emerged in an HCV-positive patient, indicating a random occurrence that may be impacted by viral causes. However, their low frequency and lack of correlation with illness stage or prognosis indicate a restricted carcinogenic involvement. This is consistent with a prior research that revealed that NRAS mutations were discovered at a higher frequency in H/L patients (2.9%) than in NHW patients (1.3%), however this difference was not significant (*p* = 0.2669) [21].

This investigation revealed that AKT1 mutations were more prevalent and varied than NRAS mutations, with the recurrent p.Asp32Glu variation identified in several clinical groups. Additional polymorphisms, such as p.Tyr26Cys and p.Pro24=, were common among HCV-infected individuals and genomic Group A, indicating a potential interplay between viral infection and host genetic predisposition. The identification of AKT1 mutations at all clinical stages corroborates the concept that the deregulation of the PI3K/AKT pathway is an early and enduring occurrence in hepatocarcinogenesis.

Previous studies provide mechanistic evidence that AKT1 has a functional role in enhancing tumor cell proliferation and suppressing apoptosis in hepatocellular carcinoma (HCC). AKT1 has demonstrated these effects by directly regulating phosphatase and tensin homolog (PTEN) and Notch1, both of which are essential regulators of cell survival and proliferation. These findings correspond with our mutational data and underscore the oncogenic significance of AKT1 in the genesis and progression of HCC [22].

Tumor venous compression has been identified as an independent predictor of microvascular invasion and satellite nodules in HCC, highlighting the role of vascular interactions and microenvironmental stress in promoting tumor aggressiveness. These observations complement our findings on FGFR1 and CDKN2A polymorphisms, suggesting that both molecular alterations and structural changes contribute to HCC progression and recurrence [23].

Recent evidence highlights the prognostic value of vascular biomarkers in HCC. Preoperative portal venous coefficient (PVC) and hepatic arterial coefficient (HAC) have been shown to independently predict overall and recurrence-free survival after hepatectomy, where lower PVC and higher HAC are associated with poorer outcomes. These findings further support the role of vascular remodeling and hemodynamic changes, alongside molecular alterations such as FGFR1 and CDKN2A polymorphisms, in influencing HCC progression and prognosis [24].

### Stage-specific distribution of NRAS and AKT1 mutations

NRAS mutations were consistently observed across early, intermediate, and late-stage disease, suggesting they may signify initial, non-progressive molecular events in hepatocarcinogenesis. Their rarity and absence of clinical correlation limit their application as prognostic markers.

AKT1 mutations showed stage-dependent enrichment, with the highest incidence in intermediate-stage HCC (50%), followed by late-stage (33.3%) and early-stage (16.7%). This pattern shows that they accumulate throughout tumor evolution, potentially contributing to increased aggressiveness, resistance to therapy, and a poor prognosis.

The distribution of AKT1 mutations corroborates previous research connecting PI3K/AKT pathway activation with suboptimal therapeutic responses and negative outcomes. Comparable patterns have been identified in other malignancies, including invasive lobular breast carcinoma, where actionable mutations in AKT1, HER2, and HER3 are prevalent [25]. These data underscore AKT1 as a prospective marker of progression and treatment resistance across various tumor types.

### Low-impact NRAS and AKT1 variants

A conservative AKT1 alteration (p.Asp32Gly, rs201636005) and a synonymous NRAS mutation (p.Asp47=, rs901561299) were shown to have modest anticipated functional impact. These may nevertheless have an impact on mRNA stability or splicing, particularly in regulatory or conserved areas, even when they may not substantially change protein structure.

Their presence in central oncogenic pathways indicates a potential role in modulating tumor heterogeneity or cooperating with other mutations, thus necessitating their inclusion in comprehensive genomic profiling.

### Predicted functional impact of mutations

This study identified six missense mutations in the AKT1 gene, specifically the recurrent p.Asp32Glu (rs201636005), p.Asn31Ile (rs780173607), and p.Glu40Gly (rs2140949199). The variants situated at conserved residues within the kinase domain may influence AKT1 activity, although their predicted effects vary. Comparable results in a cohort of Jordanian prostate cancer patients indicated the presence of multiple missense mutations in exon 4 [26]. These results underscore the importance of non-hotspot AKT1 mutations in tumorigenesis and highlight the need for broader mutational profiling in HCC.

A somatic SNV at position chr14:105246481 (c.119 A > G) in the AKT1 gene was identified, leading to a p.Glu40Gly missense mutation. This variant, formerly designated as rs2140949199, was identified in various AKT1 transcripts and exons, specifically ENST00000402615.2 and ENST00000349310.3. This has been documented in HCC and other malignancies. Functional prediction tools suggested tolerability by SIFT (0.1) and potential damage by PolyPhen-2 (0.597), indicating a moderate overall impact. The uncertain results from FATHMM and MutationTaster, coupled with its presence in a conserved region, indicate potential functional relevance in HCC biology.

NRAS mutations—p.Asp47Glu and p.Asp47Ala—were also predicted to be deleterious or disease-causing by multiple tools this data aligns with a previous study that it is reported Variants such as *KRAS* G12S, E49K, and *NRAS* R102Q were predicted as benign by CancerVar but pathogenic by others (PrimateAI, BayesDel_addAF, REVEL, FATHMM-MKL, CADD_Phred) [27]. The p.Asp47Ala variant in particular may impair protein function due to the loss of a negatively charged residue, potentially affecting RAS-MAPK signaling.

Beyond molecular and vascular factors, recent studies have identified PANoptosis, a novel form of programmed inflammatory cell death, as a key regulator of HCC progression, immune modulation, and therapy resistance. This mechanism represents a promising prognostic and therapeutic target, complementing our findings on the role of genetic alterations in HCC aggressiveness [28].

This study has some limitations that should be acknowledged. The relatively small number of HCC patients (*n* = 21) may limit the statistical strength and generalizability of the results. In addition, the analysis was based on a targeted gene panel, which focused on selected cancer-associated regions and may not capture the full spectrum of genetic alterations involved in hepatocarcinogenesis. Therefore, further studies with larger, multi-center cohorts and comprehensive genomic approaches, such as whole-exome or whole-genome sequencing, are recommended to validate and expand upon our findings in the Egyptian population.

## Conclusion

This study provides novel insights into the mutational patterns of the AKT1 and NRAS genes in Egyptian patients with hepatocellular carcinoma (HCC). NRAS mutations were uncommon and evenly distributed across disease stages, whereas AKT1 mutations were more common and enriched in intermediate and late-stage HCC, indicating a probable role in disease development. The frequent finding of the p.Asp32Glu variation, combined with additional mutations affecting conserved areas of the kinase domain, emphasizes AKT1’s functional role in carcinogenesis. The presence of particular AKT1 mutations in HCV-infected individuals suggests a link between viral infection and genetic vulnerability. Despite forecasts of modest impact, the existence of some variations within important oncogenic pathways such as PI3K/AKT and RAS/MAPK emphasizes the importance of complete profiling. The findings suggest that AKT1 could be used as a biomarker for progression and a target for HCC treatment.

## Data Availability

Due to privacy and patient identification concerns, the datasets generated and/or analyzed during the current study are not publicly available. However, they are available from the corresponding author upon reasonable request at [Mohamed.khalifa@57357.org](mailto: Mohamed.khalifa@57357.org) . This has been clearly stated in the Data Availability section of the manuscript and entered in the submission system.
